# Thermally Stable Collagen from Black Carp (*Mylopharyngodon piceus*) Swim Bladder: Preparation, Structure, Rheological, and Functional Properties

**DOI:** 10.3390/foods14193359

**Published:** 2025-09-28

**Authors:** Lichi Wei, Yushuang Li, Cong Ke, Junde Chen, Jing Zhang

**Affiliations:** 1 Fisheries College, Jimei University, Xiamen 361021, China; 15277848229@163.com; 2 Technical Innovation Center for Utilization of Marine Biological Resources, Third Institute of Oceanography, Ministry of Natural Resources, Xiamen 361005, China; liyushuang@tio.org.cn (Y.L.); 13172508013@163.com (C.K.)

**Keywords:** black carp, swim bladder collagen, structure, rheological properties, functional properties

## Abstract

Fish-derived collagen can reduce the risk of disease transmission and has no religious or cultural restrictions. However, it has limited applications due to its poor thermal stability. In this study, black carp swim bladder collagen (BBC), classified as a type I collagen, was extracted. Amino acid composition analysis revealed that BBC had a higher proline hydroxylation rate of 39.57%. Fourier transform infrared spectroscopy revealed that BBC exhibited a complete triple-helix structure. The fractional viscosity curve and differential scanning calorimetry curves revealed that the thermal denaturation temperature (Td) and the melting temperature (Tm) were 30.85 °C and 107.19 °C, respectively. The dynamic rheological analysis showed that as the concentration increased from 5 mg/mL to 20 mg/mL at 0.01 Hz, the storage modulus increased from 0.979 Pa to 84.2 Pa. When the temperature exceeded the Td, the BBC solution exhibited viscous behaviour as the frequency increased. The steady-shear analysis showed that the BBC was a shear-thinning fluid. Functional properties analysis revealed that BBC exhibited better emulsification properties, foaming properties, water absorption capacity and oil absorption capacity than land-derived collagen, making it suitable for emulsifiers, bubbling beverages, and frozen meat preservation. Additionally, BBC promoted the growth of MT3C3-E1 cells and maintained the normal morphology of the cells. These results showed that BBC is a promising substitute for terrestrial collagen in functional foods, cosmetics, and biofunctional materials.

## 1. Introduction

Collagen is an important protein found in the extracellular matrix of living organisms and is mainly located in tissues, particularly in skin, tendons, and bones [1]. Currently, at least 29 types of collagen have been identified. Among all of the discovered collagen types, type I collagen is the most predominant collagen type in animal organisms. Owing to its high biodegradability and biocompatibility of collagen, it has a wide range of applications in multiple fields, including food processing, pharmaceutical research and development, and cosmetics production [2]. In 2020, the global collagen market was $4.7 billion. The collagen market has been predicted to grow further by 2027, reaching $7 billion [3]. However, international trade of terrestrial collagen is limited by several factors, including religious and cultural taboos and infectious disease risk (e.g., bovine spongiform encephalopathy and swine fever) [3,4]. In contrast, fish-derived collagen has attracted considerable attention from researchers owing to its high safety, low immunogenicity, and freedom from cultural or religious restrictions.

However, due to their long-term aquatic habitats, the thermal stability of collagen is significantly lower than that of terrestrial animals. For example, the thermal denaturation temperatures of chum salmon skin collagen, deep-sea redfish skin collagen, and Pacific cod skin collagen are 12.01, 16.1, and 14.5 °C, respectively [3,5,6]. The poor thermal stability of fish-derived collagen has become a major challenge restricting the development and market promotion of its high-value-added products. Therefore, developing fish collagen with good thermal stability is urgent and desirable.

The rheological properties of collagen are important processing indicators. Regulating the rheological properties enables the production of products with high solid content, good system stability, low viscosity, and uniform flow properties [7]. In the cosmetics industry, consumers particularly prefer products with good spreadability, a characteristic associated with shear-thinning behaviour [8]. Relevant studies have been conducted on the rheological properties of collagen. Gao et al. found that collagen solutions derived from double-spotted pufferfish skin exhibited shear-thinning behaviour. Additionally, they found that the viscosity of the collagen solutions changed with concentration and temperature [8]. He et al. conducted a comparative study on the rheological properties of terrestrial-sourced collagen and fish-sourced collagen. They found that under the same experimental conditions, the elastic behaviour of pig skin collagen was more pronounced than that of fish-sourced collagen, providing a reference for the research on rheological differences among collagen from different sources [9]. Similarly, Li et al. analysed the influence mechanisms of three key factors, including concentration, molecular size, and temperature, on the rheological behaviour of collagen solutions. The findings provide important theoretical support for research and applications related to collagen processing [7].

The functional properties of collagen determine its applicability for different fields. Some studies have been conducted on the exploration of the functionality of collagen. Guan et al. investigated the functional characteristics of silver carp collagen, including emulsification, foaming, water absorption capacities, and oil absorption capacities. The result shows that the collagen has potential applications in food and cosmetics [10]. Li et al. found that collagen derived from grass carp swim bladders exhibited better foaming and emulsifying properties than terrestrial sources, making it suitable for producing food-grade emulsifiers and foaming agents [11]. Zheng et al. analysed the functional properties of chum salmon skin collagen and Nile tilapia skin collagen, providing insights into the suitability of different collagen sources for various applications [3].

Tilapia, grass carp, and black carp (*Mylopharyngodon piceus*) are the main economic freshwater fish in China, and they are omnivorous, herbivorous, and carnivorous fish, respectively. Black carp lives in the bottom water layer for a long time and has strong activity ability [12,13,14]. The black carp is a good source of protein due to its lifestyle. According to the ‘China Fisheries Yearbook’, the total production of black carp in China was approximately 800,000 tons in 2023, which was 6.96% higher than that produced in 2022, with resource levels remaining stable [15]. A processing byproduct of black carp, the swim bladder is rich in collagen and is thus a promising source of collagen. However, key properties that determine its practical value, including thermal stability, rheological behaviour, and functionality, have not been characterised for collagen from this material.

In this study, collagen was extracted from the black carp swim bladder. The structural features, thermal stability, rheology, and functionality of the extracted collagen were analysed. This study aimed to assess the suitability of black-carp-based collagen as a sustainable replacement for conventional land-based collagen in different applications, including food processing, cosmetic, and biofunctional materials.

## 2. Materials and Methods

### 2.1. Materials

Swim bladders of black carp (*Mylopharyngodon piceus*) were sourced from Beihai Quality Aquatic Products Co., Ltd. (Beihai, China). The 4× Laemmli Sample Buffer was produced in Bio-Rad Laboratories (Cat. No. 1610747, Hercules, CA, USA). Protein marker was sourced from Thermo Fisher Scientific Baltics (Cat. No. 26634, Vilnius, Lithuania). Type I collagen derived from rat tail was sourced from Sigma Chemical Company (St. Louis, MO, USA). MC3T3-E1 cell lines were sourced from Cobioer (Cat No. CBP60946, Nanjing, China). Acetonitrile, of HPLC grade, along with other analytical-grade reagents, were used in the experiments.

### 2.2. Preparation of Collagen from Swim Bladders of Black Carp

Collagen was extracted following the procedure reported by Guan et al. [10]. After excess fat was removed from the swim bladder, the tissue was dissolved in 0.5 M acetic acid (1:70 *v*/*v*). Following thorough mixing, the solution was centrifuged at 9000 rpm for 30 min at 4 °C (Avanti J-26 XP, Beckman Coulter, Brea, CA, USA). After the supernatant was collected, sodium chloride was added to it until the final concentration of sodium chloride in the solution was 4% (*w*/*v*). After centrifugation, the resulting precipitate was thoroughly stirred in a 0.5 M acetic acid solution at a 1:10 (*w*/*v*) ratio, then transferred to a dialysis bag (MD 77 MM, Viskase, Darien, IL, USA). The dialysis was performed using a 0.1 M acetic acid solution for 24 h, followed by continued dialysis with pure water for 48 h. After dialysis, the collagen solution was freeze-dried (LyoBeta-25, Telstar, Terrassa, Barcelona, Spain).

(1) $$Yield\;(\%)=\frac{M}{M_{0}}\times 100,$$

where *M*_0_ refers to the weight of dried fish swim bladder (g), and *M* refers to the weight of freeze-dried collagen (g).

### 2.3. Structural Characterisation

#### 2.3.1. Sodium Dodecyl Sulphate–Polyacrylamide Gel Electrophoresis (SDS–PAGE)

Electrophoresis of BBC was performed following the reported procedure of Gao et al. [8]. A 1 mg/mL collagen solution was diluted 1:3 (*v*/*v*) with 4× Laemmli sample buffer, then heated in a metal bath at 100 °C for 5 min. The electrophoresis gel consisted of a 4% stacking gel and a 6.5% separating gel. Protein standards (26634), rat tail type I collagen, and the sample were applied to the gel (10 μL), and electrophoresis was performed using an electrophoresis apparatus (Bio-Rad Laboratories, Hercules, CA, USA) at a constant voltage of 110 V. After electrophoresis, the gel was treated with a fixing solution for 30 min, followed by staining for 30 min, and finally decolorization for 20 min. Protein molecular weight analysis was performed using Tanon Image software (Version 4.0, Tanon Science & Technology Co., Ltd., Shanghai, China). The grayscale ratio of the band was determined using Image J (Version 1.8.0, National Institutes of Mental Health, Bethesda, MD, USA).

#### 2.3.2. Amino Acid Composition

Amino acid compositions in BBC were measured according to a method reported by Chen et al. [16]. The samples were digested at 110 °C for 21 h and then filtered. Sodium hydroxide was added to the filtrate after nitrogen purging. After ultrasonic treatment, derivatisation treatment was performed. Finally, the sample was analysed using HPLC-MS/MS (Ultimate 3000-API 4000 Q TRAP, Thermo Fisher Technologies, Dreieich, Germany) with MSLab 50AA-C18 (150 mm × 4.6 mm, 5 μm). The MS parameters were as follows: ionisation: +ESI electrospray; scan mode: multiple reaction monitoring; curtain gas (20 psi); spray voltage (5.5 kV); nebuliser gas (55 psi); auxiliary gas (60 psi); atomization temperature (500 °C).

#### 2.3.3. Ultraviolet Visible (UV) Absorption

The UV absorption experiment was conducted following the procedure reported by Chen et al. [16]. Collagen was dissolved in 0.5 M acetic acid to prepare a 0.5 mg/mL collagen solution. Absorbed spectrum was recorded between 220 and 400 nm using a spectrophotometer (UV-1780 SHIMADZU, Kyoto, Japan). The 0.5 M acetic acid was used as a blank control.

#### 2.3.4. Fourier Transform Infrared (FTIR)

The FTIR spectroscopy was performed following the method of Chen et al. [16]. Collagen and KBr were ground at a 1:100 ratio (*w*/*w*). Finally, potassium bromide was used as a background. The sample was characterised using Fourier transform infrared (VERTEX 70, Bruker, Bremen, Germany) spectroscopy. The resolution was fixed at 4 cm^−1^, and the instrument accumulated 32 scans in the wave number range of 4000–400 cm^−1^.

#### 2.3.5. FTIR Spectral Curve Fitting

The infrared spectral curves were fitted following the method proposed by Li et al. [11]. The baseline correction and smoothing of the spectrum were performed using OMNIC software (Version 8.2, Thermo Fisher Scientific Inc., Waltham, MA, USA) to obtain the spectra within the range of 1600–1700 cm^−1^. The processed spectrum was then subjected to second-derivative fitting using PeakFit software (Version 4.12, SeaSolve Software Inc., Shelton, DC, USA). The relative content of each secondary structure (expressed as a percentage) was determined based on the ratio of the total area of the fitted peaks corresponding to each secondary structure type to the total area of all fitted peaks.

#### 2.3.6. X-Ray Diffraction (XRD)

XRD was conducted following the method proposed by Chen et al. [16]. After the collagen sample was evenly placed on the sample stage, it was measured using an X-ray diffractometer (X’Pert Pro XRD, PANalytical, Almelo, The Netherlands). The scan range was 5–90° (2θ), and d-spacing was measured using the Bragg equation as follows:

(2) $$d\left(Å\right)\;=\;\frac{\lambda }{2\;sin\theta },$$

where *λ* refers to the X-ray wavelength (1.54°), and *θ* refers to the Bragg diffraction angle.

### 2.4. Zeta Potential

The BBC’s zeta potential was determined following the previously reported method of Kaewdang et al. [17]. First, 0.2 mg/mL of collagen was prepared in 0.1 M acetic acid. The pH of all of the samples was adjusted to 2, 3, 4, 5, 6, 7, 8, 9, and 10 using 1 M HNO_3_ and 1 M KOH. Zeta potentials of the samples at different pH levels were measured using a zeta potential analyser (Zetasizer Nano ZS 90, Malvern Instr., Malvern, UK).

### 2.5. Thermal Stability

#### 2.5.1. Denaturation Temperature (Td)

The Td of BBC was tested following the procedure reported by Chanmangkang et al. [18]. A cone-plate geometry (CP 25-2) (2° cone angle, 25 mm diameter) was used with a 103 μm gap distance between the upper and lower plates. Collagen (10 mg/mL) was prepared in 0.5 M acetic acid. Viscosity changes were assessed using a rheometer (MCR 302, Anton Paar, Graz, Austria) within a temperature range of 15–50 °C at a shear rate of 1 s^−1^. The collagen solution was dropped in the centre of the bottom plate, and then the solvent was coated at the edge with dimethyl silicone oil to prevent evaporation of the solvent during the measurement. Before each test, the sample was equilibrated for 5 min at the predetermined initial temperature. The fractional viscosity was calculated as follows:

(3) $$Fractional\;viscosity\;=\;\frac{A_{T}-A_{50{}^{\circ}C}}{A_{15{}^{\circ}C}-A_{50{}^{\circ}C}},$$

where *A*_15 °C_ and *A*_50 °C_ represent viscosities at 15 °C and 50 °C, respectively, and *A*_T_ is the temperature corresponding to a certain time.

#### 2.5.2. Differential Scanning Calorimetry (DSC)

DSC was performed following the method reported by Jongjareonrak et al. [19]. The collagen was solubilised in 0.01 M acetic acid (1:40 *w*/*v*). The samples (8–10 mg) were uniformly dispersed into the base of a sealed aluminium pan, and a blank aluminium pan was used as a reference. A differential scanning calorimeter (DSC2, Mettler-Toledo, Zurich, Switzerland) was used to conduct the test. Throughout the entire testing process, nitrogen was used as the purge gas. Before the test, the instrument was calibrated using indium as a standard substance. Blank baselines were collected under the same conditions as the sample tests. The testing procedure was as follows: the sample chamber was equilibrated to an initial temperature of 20 °C and heated to 140 °C at a rate of 10 °C/min. The Tm was calculated based on the peak value of the DSC transition curve.

### 2.6. Rheological Properties

#### 2.6.1. Dynamic Rheology

Dynamic rheology was performed following the procedure reported by Li et al. [11]. The test was conducted using a rotational rheometer (MCR 302, Anton Paar, Graz, Austria) equipped with a cone/plate geometry (CP 60-0.5) (cone angle: 0.5°, diameter: 60 mm). The gap between the upper and lower plates was 57 μm, and the frequency range was 0.01–10 Hz. Collagen was dissolved in 0.5 M acetic acid, and the rheological properties of the collagen solution with different concentrations (5, 10, 15, and 20 mg/mL) were tested at 20 °C. In addition, 10 mg/mL collagen was tested at temperatures of 10, 20, 30.85, and 40 °C. A strain sweep test was performed at a fixed frequency of 1 Hz with the strain range adjusted from 0.1% to 1000%. This test was conducted to determine the linear viscoelastic region (LVR) of the different collagen solutions at 20 °C. For subsequent tests, a strain of 10% was selected to ensure that the collagen structure remained undamaged. After the solution was loaded onto the sample stage, dimethyl silicone oil was used for sealing to prevent solvent evaporation. All of the samples were equilibrated at the corresponding temperature for 5 min.

#### 2.6.2. Steady Rheology

The experiment was conducted following the procedure description in Section 2.6.1. The shear rate was set from 0.1 to 100 s^−1^. The fitting of viscosity-shear rate curves was performed using the Ostwald–de Waele Model.

(4) $$\eta =K\gamma^{n-1},$$

where *n* refers to the flow behaviour index, *η* represents the shear viscosity (Pa·s), *γ* is the shear rate (s^−1^), and *K* denotes the consistency index (Pa·s^n^).

### 2.7. Functional Properties

#### 2.7.1. Emulsifying Activity Index (EAI) and Emulsifying Stability Index (ESI)

The BBC’s EAI and ESI testing methods were referenced using the method of Gao et al. [8]. The collagen solution (5 mg/mL) was prepared with distilled water. The pH of the BBC solution was adjusted to a series of predetermined values (2, 4, 6, 7.55, 8, and 10) by adding 1 M HCl and 1 M NaOH. A homogeniser (JS25, JUNRUI, Yangzhou, China) was used to homogenise the mixture at a rotation speed of 23,000 rpm for 1 min. After emulsion preparation was completed, samples were collected from the bottom layer of the emulsion at 0 min and 10 min. Then, the samples were added to the SDS solution with a mass–volume fraction of 0.1% (*w*/*v*) and underwent a 100-fold volume dilution. The 0.1% (*w*/*v*) SDS solution was used as the blank control. A UV-Vis spectrophotometer (UV-1780 SHIMADZU, Kyoto, Japan) was used to determine the absorbance value of the BBC sample at a wavelength of 500 nm. The EAI and ESI of the BBC solution were calculated through the following formula:

(5) $$EAI\left(m^{2}/g\right)\;=\;\frac{2\;\times \;2.303\;\times {\;A}_{0}\;\times \;N}{10000\times \;\varphi \;\times \;c},$$

(6) $$ESI\left(min\right)=\frac{A\times ∆t}{∆A},$$

where *N* represents the dilution factor (100), *c* is the initial concentration of the collagen solution (mg/mL), *A*_0_ is the absorbance value measured at 0 min after the completion of emulsion preparation, *A* is the absorbance value measured at 10 min after the completion of emulsion preparation; Δ*A* is *A*–*A*_0_; Δ*t* refers to 10 min, and *φ* denotes the oil phase volume fraction (0.25).

#### 2.7.2. Particle Size Distribution

The experiment was conducted following the method proposed by Zhu et al. [20]. The emulsion was prepared in accordance with the procedure outlined in Section 2.7.1. The particle size was measured using a laser particle size analyser (LS-POP(6), Omec Instruments Co., Ltd., Zhuhai, China) at 25 °C with an obscuration level ranging from 10% to 20%. The refractive indices of water and soybean oil were 1.33 and 1.47, respectively.

#### 2.7.3. Creaming Index (CI)

The experiment was conducted following the method proposed by Ma et al. [21]. The emulsion was transferred into a transparent glass bottle with a lid and stored at 4 °C for 7 days. The creaming height of the emulsion was recorded on days 0, 1, 3, 5, and 7, respectively. Additionally, photographs of the emulsion creaming were taken on days 0 and 7 to visually record the stratification. The CI was calculated as follows:

(7) $$CI\;(\%)\;=\;\frac{H_{t}}{H_{0}}\times 100,$$

where *H*_0_ represents the total height of the emulsion, and *H_t_* is the height of the serum.

#### 2.7.4. Foaming Capacity (FC) and Foam Stability (FS)

FC and FS were measured according to the method reported by Gao et al. [8]. The BBC solution (5 mg/mL) was prepared using 0.5 M acetic acid. The BBC solution was pipetted into a centrifuge tube and subjected to continuous homogenisation using a homogeniser (JS25, JUNRUI, Yangzhou, China) at a speed of 23,000 rpm for 2 min. The foam volume at 0 min after homogenization and after standing for 1 h were recorded. The FC and FS of the BBC solution were calculated as follows:

(8) $$FC\left(\%\right)\;=\frac{\;V_{1}-V_{0}}{V_{0}}\times 100,$$

(9) $$FS\left(\%\right)=\frac{V_{2}-V_{0}}{V_{1}-V_{0}}\times 100,$$

where *V*_0_ is the total volume before homogenisation (mL), *V*_1_ represents the initial foam volume after homogenisation (mL), and *V*_2_ denotes the foam volume standing for 1 h after homogenisation (mL).

#### 2.7.5. Water Absorption Capacity (WAC)

WAC was measured following the procedure reported by Chandi et al. [22]. Collagen and distilled water were added to centrifuge tubes at a 1:10 (*w*/*v*) ratio. The mixture was vortexed for 2 min and then allowed to stand for 1 h before centrifuging at 5000 rpm for 30 min. The total mass of collagen, precipitate, and centrifuge tube was recorded.

#### 2.7.6. Oil Absorption Capacity (OAC)

OAC was measured following the procedure reported by Chandi et al. [22]. Collagen and soybean oil were added to centrifuge tubes at a 1:5 (*w*/*v*) ratio. The mixture was vortexed for 2 min and then allowed to stand for 1 h before centrifuging at 5000 rpm for 30 min. The total mass of collagen, precipitate and centrifuge tube was recorded.

### 2.8. Cell Viability and Proliferation

Cell relative growth rate was determined as described by Gao et al. [8]. Collagen solutions (0, 6.25, 12.5, 25, 50, and 100 μg/mL) were prepared with distilled water and sterilised with UV light. Then, 96-well plates were used for the seeding of MC3T3-E1 cells at a density of 5 × 10^3^ cells/well and cultured for 24 h in an incubator at 37 °C with 5% CO_2_. Cellular morphology and proliferation status of MC3T3-E1 cells were observed and photographed using an inverted microscope (ECLIPSE Ts2, Nikon Corporation, Tokyo, Japan). The absorbance of the sample was measured at 490 nm using a microplate reader (SpectralMax M5, Molecular Devices, San Jose, CA, USA). The cell relative growth rate was calculated as follows:

(10) $$\mathrm{Cell}\mathrm{relative}\mathrm{growth}\mathrm{rate}\;(\%)\;=\;\frac{A_{1}-A}{A_{0}-A}\times 100\%,$$

where *A*_0_ refers to the absorbance of the control, *A*_1_ refers to absorbance of the treatment, and *A* refers to without cell group.

### 2.9. Statistical Analysis

The results were reported as mean ± standard deviation (SD). The SPSS 17.0 software (IBM SPSS Statistics, Ehningen, Germany) was used to perform analysis of variance. A *p*-value < 0.05 is considered statistically significant.

## 3. Results

### 3.1. Yield

The collagen extracted from the swim bladder of black carp (*Mylopharyngodon piceus*) achieved a yield of 78.83 ± 0.13% relative to the dry weight of the raw tissue. This high extraction efficiency is attributable to a lower degree of cross-linking of collagen molecules that reduces the intermolecular interactions of collagen molecules, thus enhancing collagen solubility and increasing collagen production yield [10]. The extraction rate of collagen from BBC was significantly higher than that from other fish species, such as bighead carp swim bladder collagen (59.00 ± 8.10%) [23], grass carp swim bladder collagen (39.20%) [11], and even Gulf corvina swim bladder collagen (69.00 ± 1.60%) [24]. This superior extraction performance with cost efficiency and process economy makes BBC a promising raw material for collagen production.

### 3.2. Structural Characterisation

#### 3.2.1. SDS–PAGE

**Figure 1:**
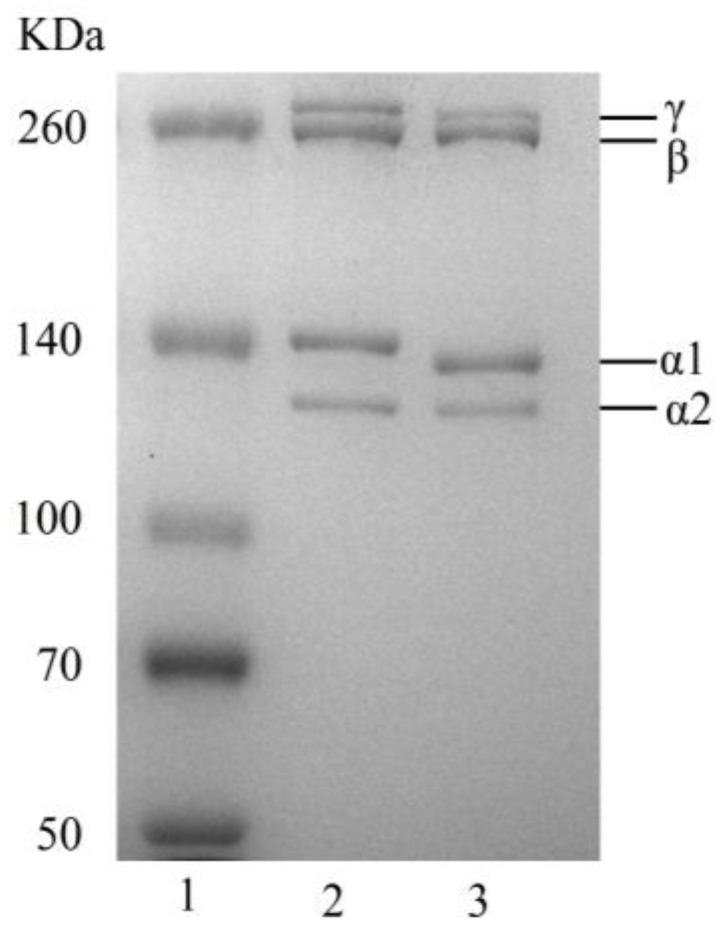
Electrophoretogram. (1) Protein Marker; (2) Rat tail type I collagen; (3) BBC.

The electrophoresis image of BBC is shown in Figure 1. The BBC and rat tail type I collagen exhibited similarity in their fundamental structural composition, with both comprising α1 and α2 chains as the primary structural units. During collagen assembly, dimeric β-chains and trimeric γ-chains were formed via intramolecular and intermolecular covalent cross-linking [3]. The contents of γ-chains and β-chains in the BBC were lower than those in rat tail type I collagen, indicating a moderate degree of cross-linking in the BBC [3]. The molecular weights of the α1 and α2 chains of BBC were approximately 134 kDa and 124 kDa, respectively, whereas those of the α1 and α2 chains of rat tail type I collagen were 138 kDa and 126 kDa, respectively. These results align with reported results on aquatic collagen from species, including Nile tilapia [25], giant salamander [26], and balloon fish [27], indicating that the BBC belongs to type I collagen.

#### 3.2.2. Amino Acid Composition

As shown in Table 1, glycine accounts for 31.20% of the total amino acids, which is a distinctive feature of collagen that reflects its characteristic (Gly-X-Y)n repeating sequence [28]. Proline and hydroxyproline accounted for 13.90% of the total amino acid content, and the calculated hydroxylation rate was 39.57% (content of hydroxyproline/content of proline and hydroxyproline), which significantly exceeded the previously reported values for walleye pollock (33.74%), silver carp skin (36.68%) [9], and brownstripe red snapper (38.21%) [19]. Studies have shown that the thermal stability of collagen is positively correlated to both amino acid content and proline hydroxylation degree [9]. This correlation is attributed to the pyrrolidine rings of proline and hydroxyproline, which restrict the conformation of the polypeptide chain and reinforce the triple-helix structure of collagen. Therefore, the high proline hydroxylation degree of the BBC indicated superior thermal and structural stability compared with other collagen sources.

#### 3.2.3. UV

**Figure 2:**
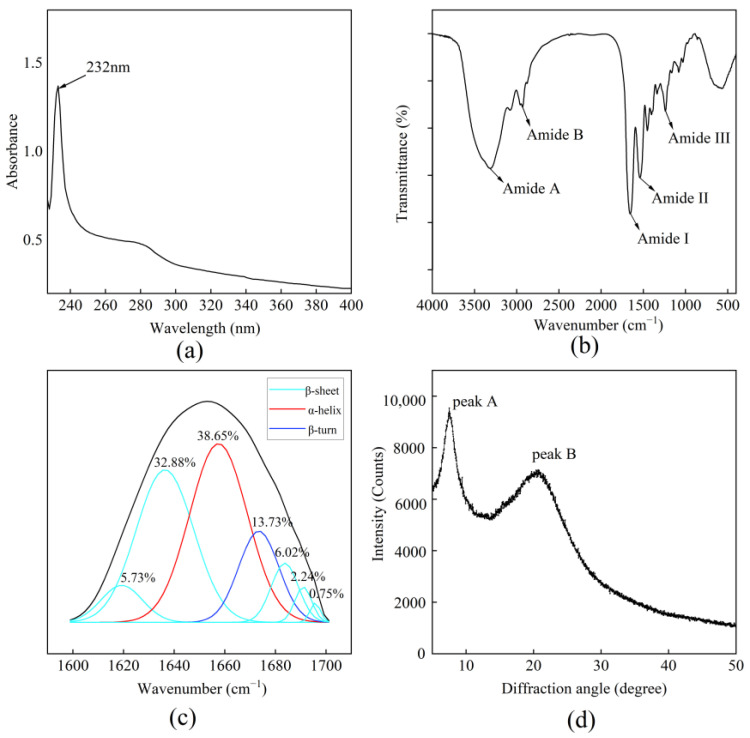
Structure of BBC. (**a**) Ultraviolet absorption spectrum; (**b**) FTIR spectrum; (**c**) Secondary structure fitting diagram. The black line refers to the FTIR spectrum in the 1600–1700 cm^−1^ range; (**d**) X-ray diffraction spectrum.

The UV absorbance spectrum of BBC is shown in Figure 2a. A significant absorption peak was observed at 232 nm, which was attributed to the presence of COOH, CO-NH_2_, and C=O functional groups in the collagen polypeptide chains [29]. A smaller spectrum peak observed at 280 nm may be attributed to phenylalanine, tyrosine, and tryptophan in collagen. This result is consistent with that of catla skin collagen (232 nm), rohu skin collagen (232 nm) [30], and tilapia skin collagen (230 nm) [31], confirming that the BBC belong to type I collagen.

#### 3.2.4. FTIR

As shown in Figure 2b, five characteristic absorption bands corresponding to amide A, B, I, II, and III were observed. The absorption peak of the amide A band was observed at 3314.64 cm^−1^, which was significantly lower than its typical range of 3400–3440 cm^−1^. This redshift indicated that the N-H groups acted as hydrogen donors and participated in the formation of intramolecular and intermolecular hydrogen-bonding networks within the collagen peptides [32]. The amide B band located at 2931.33 cm^−1^ was mainly attributed to the asymmetric stretching vibration of -CH_2_ [11]. The amide I band appeared at 1655.81 cm^−1^, within the expected region of 1600–1700 cm^−1^, and was mainly related to the C=O stretching vibration of the peptide backbone, coupled with contributions from C-N stretching and N-H in-plane bending. This band served as a key indicator for assessing the secondary structure of proteins [33]. The amide II band was detected at 1540.08 cm^−1^, which is below the conventional range of 1550–1600 cm^−1^. This frequency shift implied that the vibrational modes of C=O and N-H groups in BBC were influenced by a specific hydrogen-bonding environment. A decrease in the amide II frequency was closely related to enhanced intermolecular hydrogen bonding. The amide III band at 1238.85 cm^−1^ was mainly attributed to N-H bending vibrations [29]. Notably, the intensity ratio of the amide III band to the absorption at 1450 cm^−1^ was approximately 1.0, which was consistent with the characteristic triple-helix conformation of type I collagen, indicating that the BBC retained its native structural integrity [34].

#### 3.2.5. Infrared Secondary Structure Fitting

In the infrared spectroscopic analysis of protein structure, the amide I band (1600–1700 cm^−1^) was widely recognised as the most informative region for characterising secondary structural elements. Specific vibrational features within this region are assigned to canonical conformations: α-helix (1650–1660 cm^−1^), β-sheet (1610–1642 cm^−1^ and 1680–1700 cm^−1^), random coil (1642–1650 cm^−1^), and β-turn (1660–1680 cm^−1^) [3]. As shown in Figure 2c, the α-helix content in the BBC was observed at 1658 cm^−1^, accounting for 38.65% of the total secondary structure content. The α-helix proportion in the BBC exceeded that in the reported for collagen from chum salmon skin collagen (36.55%) [3], deep-sea redfish skin collagen (22.60%) [5], and grass carp skin collagen (19.06%) [35]. The α-helix formed a compact right-handed helix conformation stabilised by hydrogen bonds between peptide bonds, facilitating the structural stability of proteins. Therefore, the high α-helix content in BBC indicated a more stable secondary structure [8]. The β-sheet components in the BBC are represented by multiple absorption bands centred at 1619, 1636, 1683, 1691, and 1696 cm^−1^, accounting for 47.62% of the total secondary structure content. This value exceeded that of deep-sea redfish skin collagen (43.50%) [5], Nile tilapia skin collagen (39.56%) [3], and red drum fish scale collagen (35.00%) [36]. Protein hydrophobicity was negatively correlated with the proportion of β-sheet structure, indicating that the BBC had lower surface hydrophobicity, which might improve colloidal dispersibility in aqueous environments [3]. The β-turn was observed at 1673 cm^−1^, accounting for 13.73% of the total secondary structure content, while no random coil (0%) was detected. β-turn and random coil structures were involved in the processes of protein unfolding, dissociation, and rearrangement [3]. The ‘ordered’ secondary structure (α-helix and β-sheet at 86.27%) of BBC was significantly greater than the ‘disordered’ secondary structures (β-turn and random coil at 13.73%). This disparity indicated that α-helix and β-sheet were the predominant secondary structures in BBC and that BBC had a tight secondary structure [11].

#### 3.2.6. XRD

As shown in Figure 2d, the BBC exhibited two collagen characteristic peaks at diffraction angles (2θ) of 7.49° and 20.66°. The interchain distance corresponding to peak A was calculated to be 11.32 Å, and the collagen skeleton distance at peak B was 4.29 Å. Studies have shown that peak A correlates with the triple-helix structural characteristic of collagen. As the hydrogen-bonding forces between the molecules weaken, the d value increases. Peak B indicated the distance between the skeletons of collagen [8]. Notably, the collagen skeleton distance in the BBC was higher than that previously reported for Pacific cod skin collagen (3.96 Å) [6], lizardfish scales collagen (4.18 Å) [16], and yellowfin tuna skin collagen (4.1 Å) [29]. These results showed that the BBC had higher potential applicability in biofunctional materials [37].

### 3.3. Zeta Potential

**Figure 3:**
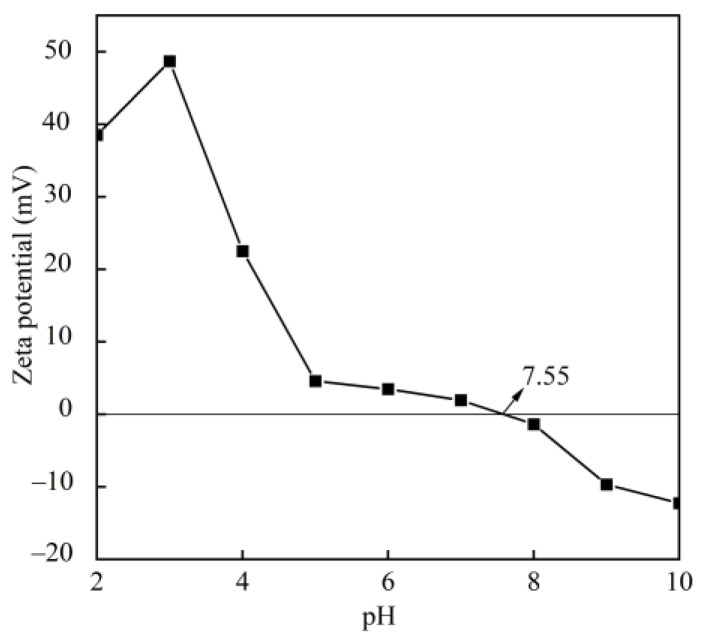
Zeta potential of BBC.

At pH 7.55, BBC exhibited an almost zero net surface charge (Figure 3), which is recognised as the isoelectric point (pI) of the collagen [17]. In the pH range of 2–7.55, the BBC was positively charged due to the dissociation of carboxylic acid or acidic side groups through their interaction with H^+^. In the pH range of 7.55–10, the BBC was negatively charged due to the dissociation of the amino or basic side groups through their interaction with OH^−^ [38]. The pI of BBC (7.55) was higher than that of others reported for other aquatic collagen sources, including grass carp swim bladder (6.59) [11], yellowfin tuna swim bladder (6.05) [17], and seabass swim bladder (6.64) [33]. The higher pI of the BBC was related to the amino acid sequence and amino acid residue distribution [37].

### 3.4. Thermal Stability

**Figure 4:**
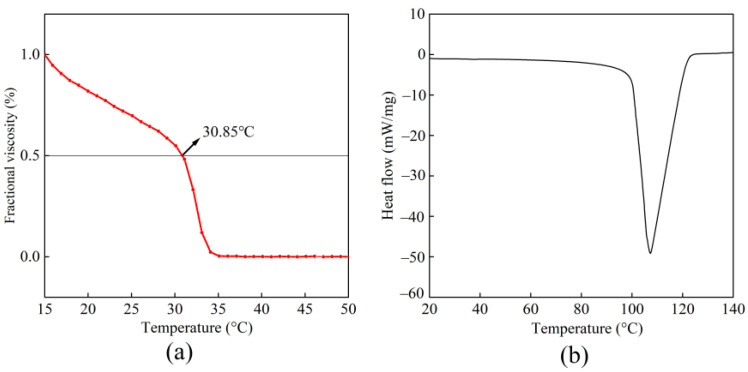
Thermal stability of BBC. (**a**) The thermal denaturation curve; (**b**) The DSC curve.

The thermal denaturation temperature (Td) is the temperature at which the relative viscosity of a collagen solution declines to half of its maximum value on the fractional viscosity curve, while the melting temperature (Tm) refers to the temperature at which collagen transforms from a solid state to a liquid state [37]. As shown in Figure 4a, the Td of BBC was 30.85 °C. When the temperature exceeded 30.85 °C, the fractional viscosity of the BBC solution sharply decreased. The energy provided by the high-temperature environment disrupted the binding force of intermolecular hydrogen bonds, causing the triple-helix structure to dissociate and gradually transform into a disordered random coil structure [32]. Comparison of the Td value of BBC with that of collagen from other reported sources revealed that its Td value (30.85 °C) was significantly greater than deep-sea redfish skin collagen (16.1 °C) [5], walleye pollock skin collagen (24.6 °C) [39], brownstripe red snapper skin collagen (30.52 °C) [19], and even exceeded human placental collagen (28.5 °C) [40]. As shown in Figure 4b, the Tm value of BBC was 107.19 °C, exceeding that of duck bone collagen (92.48 °C), duck skin collagen (86.22 °C), and duck tendon collagen (88.46 °C) [41]. The high Td and Tm values of BBC were mainly related to its proline hydroxylation rate [9]. These results are consistent with the results of amino acid analysis. The proline hydroxylation rate of BBC was 39.57% (content of hydroxyproline/content of proline and hydroxyproline). In contrast, the proline hydroxylation rates of deep-sea redfish skin collagen, walleye pollock skin collagen, and brownstripe red snapper skin collagen were 38.10, 37.5, and 38.21%, respectively. A relatively high hydroxylation level rate facilitated the formation of a denser hydrogen bond network between collagen molecules and enhanced the structural stability of the molecular skeleton [5]. In addition, the contents of the α-helix and β-sheet were also one of the factors affecting thermal stability. The main secondary structure of BBC comprised α-helix and β-sheet (accounting for 86.27%). These structures might facilitate the formation of intermolecular hydrogen bonds and a more compact triple-helical conformation, thereby enhancing thermal stability and increasing the temperature required for degradation [42]. Therefore, BBC exhibited relatively high thermal stability, indicating that it was not easily denatured during room-temperature processing and maintained the natural biological activity of collagen, thus possessing good application potential in the field of biofunctional materials [43].

### 3.5. Rheological Properties

#### 3.5.1. Dynamic Rheology

**Figure 5:**
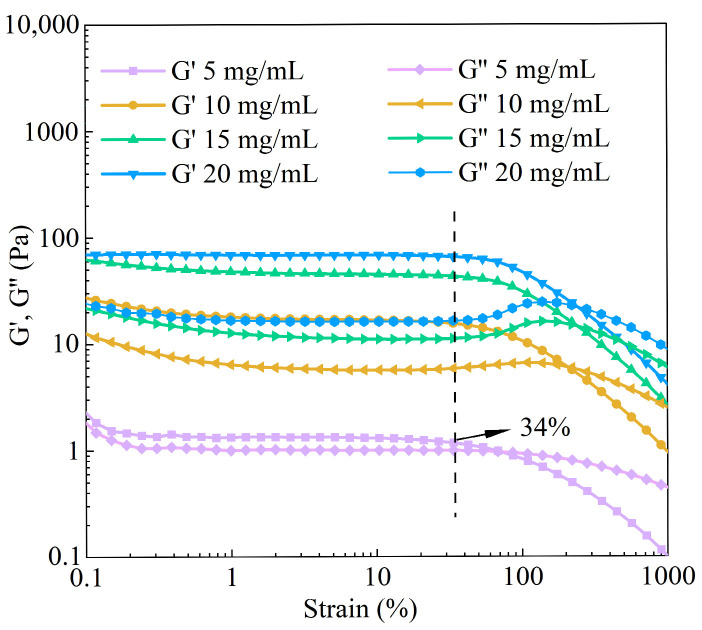
The effect of stress on the storage modulus (G′) and loss modulus (G″) of BBC.

**Figure 6:**
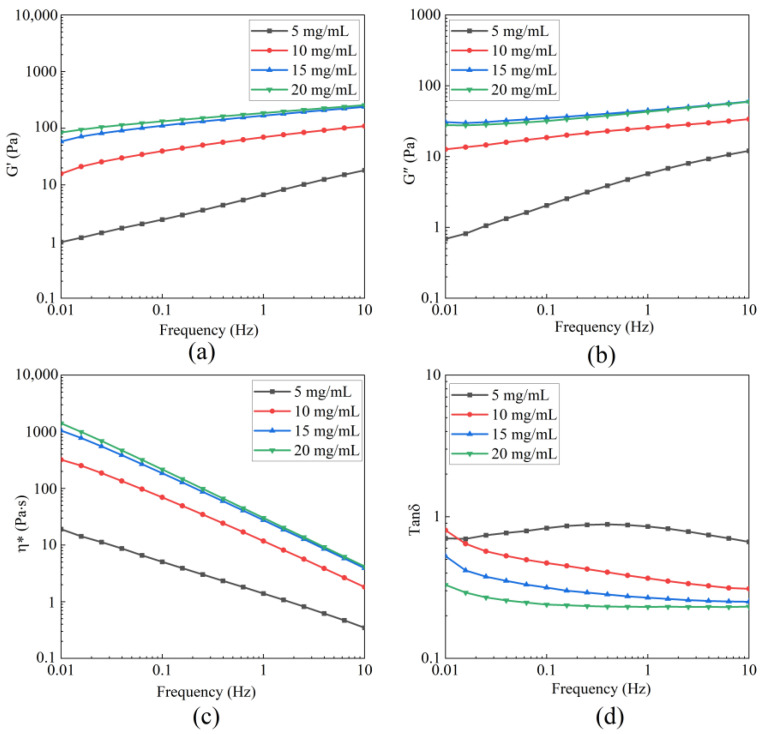
Dynamic rheological curves of BBC solution with different concentrations at 20 °C. (**a**) The storage modulus G′; (**b**) The loss modulus G″; (**c**) The complex viscosity η*; (**d**) Loss tangent (tan *δ* = G″/G′).

The LVR of BBC solutions with different concentrations was determined through strain sweep tests (Figure 5). The critical strain was 34%. Below the critical strain, storage modulus (G′) and loss modulus (G″) remained stable. When the strain exceeded the LVR, the values of G′ and G″ for collagen solutions of various concentrations were unstable and decreased, which indicated that the strain disrupted the entangled network structure of collagen [44]. The dynamic rheological results are shown in Figure 6. Under the condition of constant frequency, as the concentration of the BBC solution increased, its G′, G″, and η* all consistently increased. This result revealed concentration as an important factor regulating the rheological behaviour and viscoelastic characteristics of the BBC solution. When the frequency was fixed at 0.01 Hz, during the process of increasing the concentration from 5 mg/mL to 20 mg/mL, the value of G′ significantly increased from 0.979 Pa to 84.2 Pa. The value of G″ increased from 0.689 Pa to 27.8 Pa, and the η* increased from 19.1 Pa·s to 1410 Pa·s. The changes in G′ were more pronounced than those in G″, indicating that the enhancing effect of increased concentration on the elasticity of the BBC solution was pronounced than its effect on the viscosity. The entangled network of BBC relied on the characteristic (Gly-X-Y)n repeating sequence, where glycine enhanced the compactness of the chains, while proline and hydroxyproline formed hydrogen bonds through their pyrrolidine rings [45]. As the BBC solution concentration increased, the entangled network structure became denser, enabling it to store more energy, thereby increasing the G′. Additionally, as the friction between molecular chains increased, the solution required greater resistance to flow, which consequently enhanced the η* [7,46,47].

The loss factor tan *δ* (G″/G′) is used to assess the viscous-elastic behaviour [47]. When tan *δ* > 1, the solution exhibits viscous behaviour; conversely, when tan *δ* < 1, the solution shows elastic behaviour. As shown in Figure 6d, within the frequency range, the tan *δ* values of BBC solutions with different concentrations were consistently less than 1. This phenomenon indicated that BBC solutions always exhibited elastic behaviour [3]. Furthermore, as the concentration increased, the tan *δ* values gradually decreased, indicating that the elastic behaviour of the solution was enhanced with increasing concentration. This phenomenon is attributed to an increase in the number of molecules per unit volume in high-concentration collagen solutions, resulting in the physical entanglement network formed by molecular chains through hydrogen bonding and hydrophobic interactions becoming denser. This effect further reduced solution flow and strengthened elastic behaviour [7,47]. The enhancement of elastic behaviour was also related to the content of α-helix and β-sheet in collagen. Notably, a higher content of β-sheet increased the number of hydrogen bonds between peptide chains. The formation of the stable structure enhanced gel strength, which was consistent with the results of the secondary structure analysis of BBC [48]. The elastic properties of high-concentration systems met the requirements for texture, mouthfeel, and stability in food processing. Notably, BBC solutions exhibited elastic effects at a low concentration of 5 mg/mL. This effect indicated that the energy consumption required to achieve elastic behaviour during food processing was reduced, minimising the raw material cost in food production and making it particularly suitable for large-scale industrial production.

**Figure 7:**
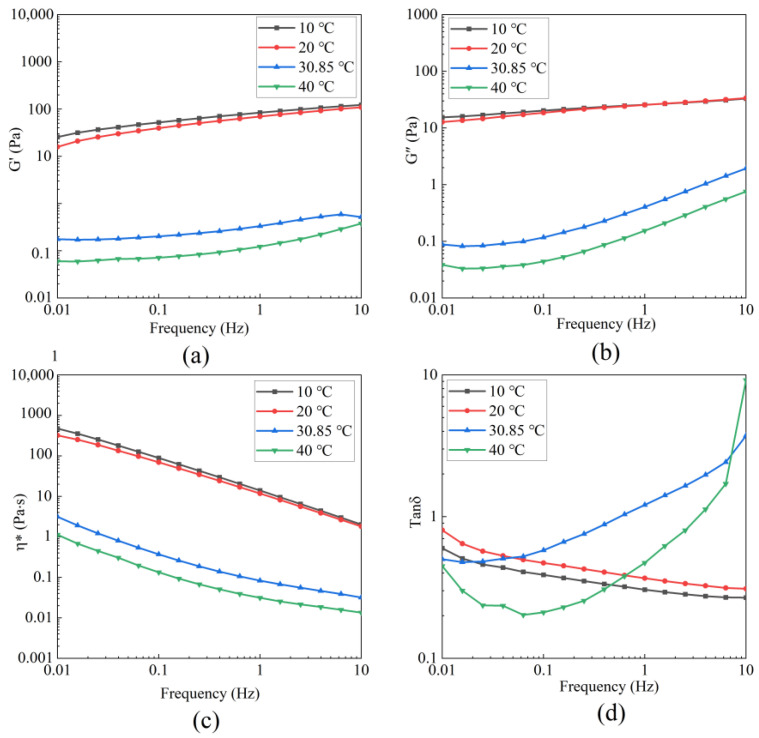
Dynamic rheological curves of 10 mg/mL BBC solution at different temperatures. (**a**) The storage modulus G′; (**b**) The loss modulus G″; (**c**) The complex viscosity η*; (**d**) Loss tangent (tan *δ*).

As shown in Figure 7, within the frequency range of 0.01–10 Hz, the values of G′, G″ and η* decreased with increasing temperature, indicating that temperature significantly affected the viscoelasticity of the solution. At 10 °C and 20 °C, the tan *δ* values of BBC remain less than 1 across the entire frequency range, indicating that the solution was dominated by elasticity. However, after the temperature reached Td, the tan *δ* values transitioned from less than 1 to greater than 1 with increasing frequency, indicating a transformation of the BBC solution from an elastic-dominated state to a viscous-dominated state. This phenomenon can be attributed to the structural disruption of collagen molecules under high-temperature conditions. Notably, the triple-helical structure of collagen unwound into random coils, which weakened the intermolecular forces. As a result, the fluidity of the solution increased, enabling the system to relax within a shorter time frame [8]. At a low temperature, the BBC showed a gel-like state dominated by elasticity, which met the requirement for system stability in yogurt production [49]. At a high temperature and low frequency, the BBC also exhibited an elasticity property. When the BBC was introduced to meat processing (e.g., sausage making), it maintained the elastic texture of sausages and reduced water loss during the cooking process [3]. These characteristics make BBC promising in the field of food processing.

#### 3.5.2. Steady-Shear Rheology

**Figure 8:**
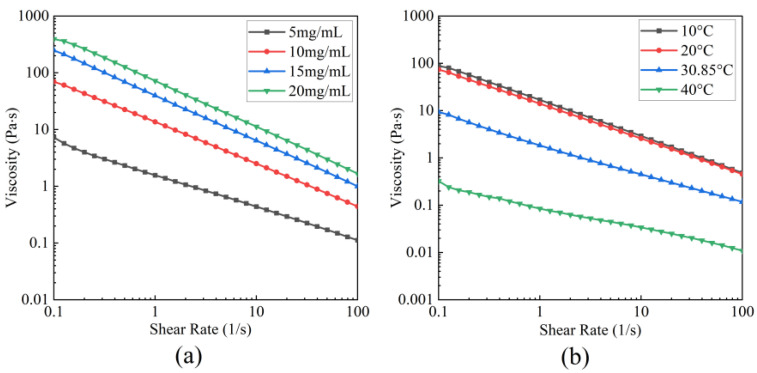
Steady state rheological curves of BBC. (**a**) Steady state rheological curves of BBC solutions with different concentrations at 20 °C; (**b**) Steady state rheological curves of 10 mg/mL BBC solution at different temperatures.

Figure 8a presents the rheological curves of the viscosity of BBC solutions with different concentrations (5–20 mg/mL) of shear rate under a constant temperature of 20 °C. As the BBC concentration increased from 5 mg/mL to 20 mg/mL at a shear rate of 0.1 s^−1^, the solution viscosity gradually increased. This phenomenon was attributed to the increased entanglements chain, which provides higher initial shear resistance, ultimately increasing viscosity [8]. Regardless of the BBC concentration, as the shear rate increased from 0.1–100 s^−1^, the solution viscosity consistently decreased. These phenomena indicated that BBC solutions with different concentrations exhibited a typical shear-thinning behaviour, a characteristic of pseudoplastic fluids [50]. The essence of this behaviour is that high shear forces disrupt the original entanglement network between molecular chains, causing the molecular chains to align directionally along the shear direction. This alignment reduces the internal frictional resistance during flow, thereby decreasing viscosity [3,47]. The Ostwald–de Waele model was used to fit the experimental data. The results showed that the goodness-of-fit index *R*^2^ exceeded 0.9 for all concentrations. As shown in Table 2, the *n* values of the BBC solutions are less than 1, confirming that BBC solutions are typical pseudoplastic fluids with shear-thinning properties. Furthermore, as the concentration increased, the consistency index (*K*) increased, reflecting the enhanced pseudoplastic behaviour of the BBC solution [7].

Figure 8b presents the correlation curves between viscosity and shear rate of the BBC solution at a concentration of 10 mg/mL under different temperature conditions. The apparent viscosity of the BBC solution decreased with the increase in shear rate. This phenomenon indicated that the solution exhibited a typical shear-thinning behaviour at all tested temperatures. At a shear rate of 0.1 s^−1^, the viscosity of the BBC solution was relatively high at 10 °C and 20 °C. When the temperature was Td, the viscosity significantly decreased. The increase in temperature transformed the collagen triple helix into an irregular coil, rapidly decreasing viscosity [7]. As shown in Table 3, the *n* values are less than 1, confirming that BBC solutions exhibit strong pseudoplasticity. Meanwhile, the *K* value decreased with increasing temperature, reflecting that the viscosity of the BBC solution decreased, and its shear-thinning behaviour weakened [47].

The shear-thinning behaviour plays a crucial role in the development and production of food and cosmetics [51]. In the food processing process, a high-concentration solution exhibits a low-viscosity fluid behaviour due to shear thinning, which enables uniform extrusion through moulds [52]. In the industrial production of skincare products, the reduction in viscosity under high shear conditions enables the formation of a smooth cream texture, which enhances the spreadability of the product during application [53]. After application, the viscosity rebounds to form a thin protective film, reducing the loss of active ingredients and improving the moisturising and protective effects of the skincare product [8]. Therefore, the shear-thinning behaviour of BBC is expected to be applied in the food and cosmetic fields.

### 3.6. Functional Properties

#### 3.6.1. EAI and ESI

**Figure 9:**
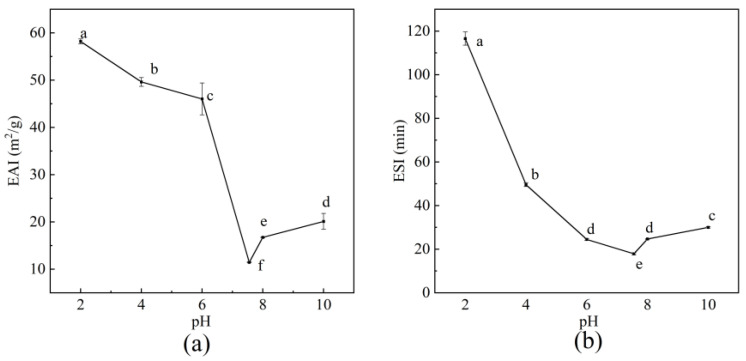
Emulsifying properties of BBC under different pH conditions. (**a**) Emulsifying activity index. (**b**) Emulsifying stability index. The labeling of different letters (a–f) indicates that there are statistically significant differences among the samples.

In food processing, emulsifying properties and emulsification stability mainly determine the texture and mouthfeel of products [54]. As shown in Figure 9, the EAI and ESI of BBC range from 58.20 ± 0.21 m^2^/g to 11.47 ± 0.38 m^2^/g and from 116.56 ± 1.24 min to 17.88 ± 0.21 min, respectively. The lowest values were achieved at the isoelectric point and a pH of 7.55. As an ampholyte, the stability of the collagen solution is determined by the amount of surface charge on the molecules. When the solution pH was close to the pI of collagen, the net surface charge of collagen gradually became zero, and the repulsive force between adjacent collagen molecules weakened, leading to aggregation. This aggregation restricted the movement and diffusion of collagen emulsion at the oil–water interface [55]. When the solution pH value was lower than the pI, the collagen emulsion was positively charged, and the acid-soluble collagen molecules adsorbed at the oil–water interface generated significant repulsive forces due to electrostatic interaction. These repulsive forces acted as a barrier, preventing the aggregation and coalescence of emulsion droplets [20]. When the pH of the solution was higher than that of the pI, the number of negative charges on the surface of protein molecules increased, and the electrostatic repulsion effect between molecules became stronger. This effect facilitated the spreadability of proteins at the oil–water interface and enhanced the emulsification effect [55]. Therefore, when collagen is used as a food emulsifier, adjusting the pH of the resulting solutions to a range significantly deviating from the pI enables the achievement of optimal emulsifying performance. The EAI and ESI values of BBC were greater than those of terrestrial collagen, including rabbit skin collagen (46.60 m^2^/g and 9.80 min), rabbit meat collagen (44.70 m^2^/g and 9.80 min), and rabbit ear collagen (48.20 m^2^/g and 9.80 min) [56]. Thus, the emulsifying properties of BBC make it an ideal choice as a substitute for terrestrial-sourced collagen in emulsification-related applications.

#### 3.6.2. Particle Size Distribution

**Figure 10:**
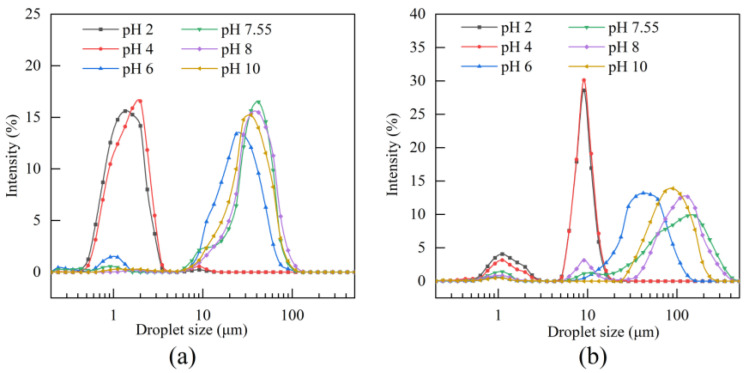
Particle size distribution of BBC emulsions under different pH conditions. (**a**) Particle size distribution on day 0. (**b**) Particle size distribution after 7 days of storage.

Figure 10 presents the particle size distributions of emulsions prepared with BBC protein under different pH conditions, measured at days 0 and 7 of storage. On day 0, emulsions at pH values of 2 and 4 exhibited the optimal emulsification performance, with particle sizes predominantly distributed within the range of 1–5 μm. This phenomenon occurred because the collagen molecules were positively charged within the above pH range, generating strong electrostatic repulsion that effectively prevented droplet aggregation, thereby facilitating the formation of fine and uniform emulsions [20]. As the pH increased, the emulsion particle size gradually increased, peaking at 41.17 μm near the isoelectric point (pH 7.55), indicating a significant decline in emulsifying capacity. After 7 days of storage, distinct differences in stability were observed among emulsions at different pH values. Emulsions at pH values of 2 and 4 maintained relatively small particle sizes (5–10 μm) with minimal change, demonstrating good physical stability. In contrast, emulsions within the pH 6–10 range significantly increased in particle size, indicating poor system stability. When pH exceeded the pI, the collagen molecules became negatively charged, and the resulting electrostatic repulsion consistently contributed to a certain degree of emulsion stability, as evidenced by the smaller particle size increase compared with the region near the isoelectric point [20]. These particle size distribution results are consistent with the results of the EAI and ESI analyses, collectively confirming that pH influences emulsion stability by regulating the protein surface charge.

#### 3.6.3. CI

**Figure 11:**
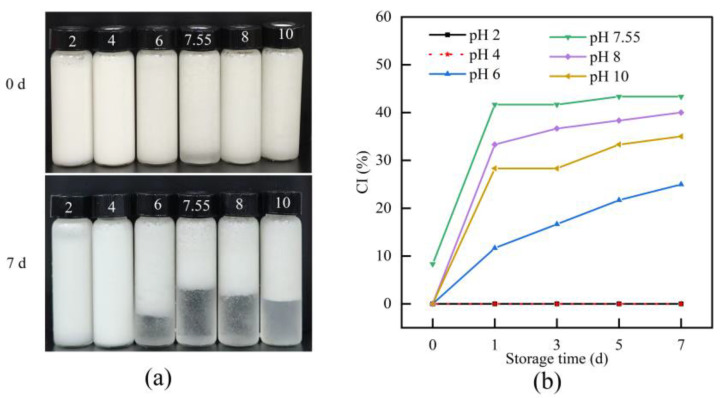
Effect of pH on the stability of BBC emulsions. (**a**) Phase separation of emulsions treated at different pH values after 0 and 7 days of storage at 4 °C; (**b**) Changes in the CI of emulsions under different pH treatments from day 0 to day 7.

Figure 11 presents the stability changes in BBC emulsions prepared under different pH conditions after 0 and 7 days of storage. Under acidic conditions (pH 2 and 4), the emulsions remained stable throughout the storage period, with no visible phase separation observed. In contrast, when the pH was within the range of 6–10, all emulsions exhibited severe phase separation, which was evident in the formation of a transparent serum layer at the bottom of the system. Notably, the phase separation was most prominent at a pH of 7.55. The CI value of the emulsion at a pH of 7.55 significantly increased from 8.33% to 43.33%, which was higher than that of emulsions prepared at other pH levels. This result showed that at a pH of 7.55, the changes in the charge amount of the system affected the BBC emulsion, significantly weakening intermolecular forces. Thus, massive aggregation of emulsion droplets were observed, ultimately resulting in poor storage stability of the emulsion [21]. These findings are consistent with the conclusions from particle size analysis, further confirming that BBC emulsions possessed excellent storage stability under acidic conditions.

#### 3.6.4. FC and FS

**Figure 12:**
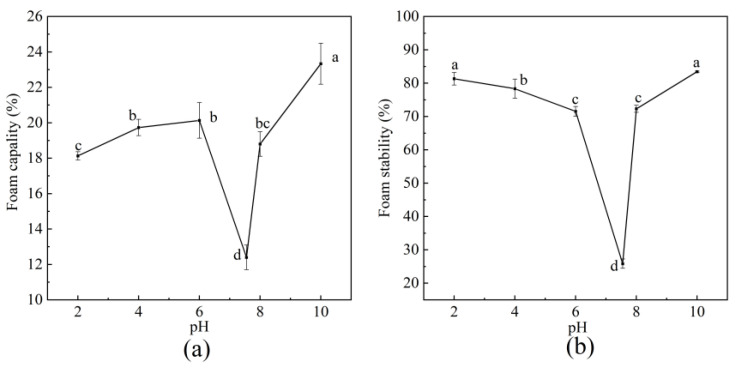
Functional properties of BBC. (**a**) Foam capacity; (**b**) Foam stability. The labeling of different letters (a–d) indicates that there are statistically significant differences among the samples.

As shown in Figure 12, both FC and FS of BBC exhibited their lowest values at pI. The overall variation range for FC ranged from 12.40 ± 0.69% to 23.33 ± 1.15%. Under the same pH conditions, the BBC exhibited a higher FC than the conventional market proteins, such as defatted cashew protein (14.00 ± 2.00%) [57], casein (3.95 ± 0.07% to 14.25 ± 0.35%), and HBC 19 rice bran protein concentrate (5.20 ± 0.28% to 10.03 ± 0.39%) [22]. The higher FC of BBC may be attributed to its solubility, protein content, and surface hydrophobicity. Under high-solubility conditions, proteins rapidly reduced surface tension and formed foam [58]. The FS of BBC ranges from 25.86 ± 1.40% to 83.43 ± 0.18%, which was higher than Basmati 370 rice bran protein concentrates (0.00% to 1.33 ± 0.05%), HBC 19 rice bran protein concentrate (3.67 ± 0.09% to 4.30 ± 0.16%), and Basmati 386 rice bran protein concentrate (0.65 ± 0.02% to 2.50 ± 0.03%) [22]. The enhanced FS can be attributed to the stability of the protein membrane formed at the gas–liquid interface and its gas permeability [3]. The BBC had higher foaming performance than other terrestrial proteins, making it an ideal choice for the production of food, carbonated drinks, beer, and other beverages.

#### 3.6.5. WAC

WAC is defined as the capability of collagen to capture water molecules on its molecular surface or inside its structure by forming multiple intermolecular interactions with water through its unique molecular structure, while preventing water loss induced by external forces (e.g., gravity, centrifugation, and heating) [8]. The WAC value of BBC was 31.12 ± 2.94 g/g, significantly surpassing those of porcine skin collagen (0.21 ± 0.03 g/g), casein (2.48 ± 0.11 g/g) [22], and cashew protein isolate (2.20 ± 0.01 g/g) [57]. The higher WAC can retain water and promote cell growth. The advantage of BBC having a higher WAC than terrestrial proteins is that it increases its application potential and development value in medical repair and cosmetic care [59].

#### 3.6.6. OAC

OAC reflects to the ability of proteins to interact with fats. In this study, the OAC value of BBC was 42.25 ± 2.81 g/g, significantly surpassing those of chicken foot collagen (5.30 ± 0.30 g/g) [60], soy protein isolate (3.49 ± 0.32 g/g) [61], and cashew nut protein isolate (4.42 ± 0.01 g/g) [57]. This performance indicated that the BBC had more prominent functional advantages than traditional terrestrial proteins. The relatively high OAC of BBC endowed it with a non-negligible application value in meat product processing. When BBC is added to meat products, it can bind to fat more efficiently and reduce juice loss during the thawing of frozen products, thus ensuring the quality and stability of meat products [62].

### 3.7. Cell Viability and Proliferation

**Figure 13:**
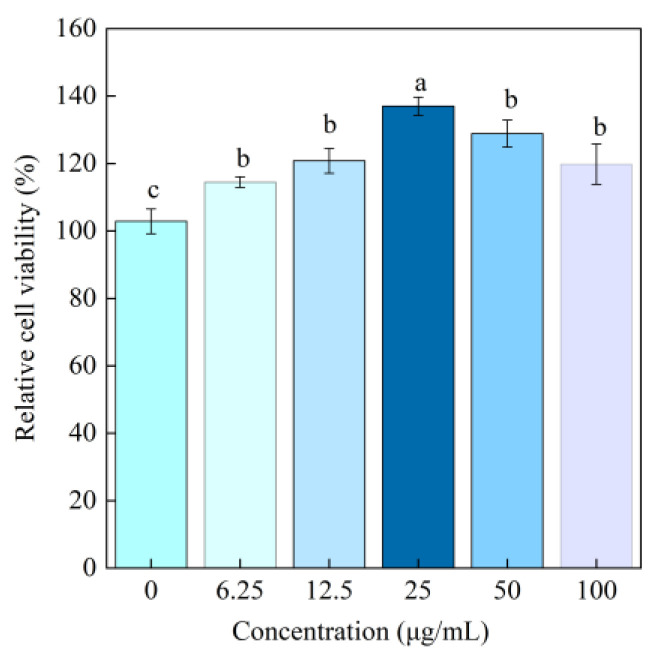
Cell relative proliferation rate after adding different concentrations of BBC for cultivation. The labeling of different letters (a–c) indicates that there are statistically significant differences among the samples.

As shown in Figure 13, the cell relative proliferation rate was 102.87% at a BBC concentration of 0 μg/mL. As the BBC concentration increased from 6.25 μg/mL to 25 μg/mL, the cell relative proliferation rate significantly increased, reaching a peak value of 136.99%. This peak value was significantly higher than that of the blank control group (*p* < 0.05). The cell relative proliferation rate in all experimental groups exceeded 100%, which was significantly higher than that of the negative control group (*p* < 0.05). According to the general evaluation standard in cell biology research, a cell viability exceeding 70% is considered nontoxic to cells [8]. In this study, within the tested concentration range of 6.25–100 μg/mL, BBC exhibited no cytotoxicity, significantly promoting cell proliferation. Therefore, BBC holds promising applications in biofunctional materials.

**Figure 14:**
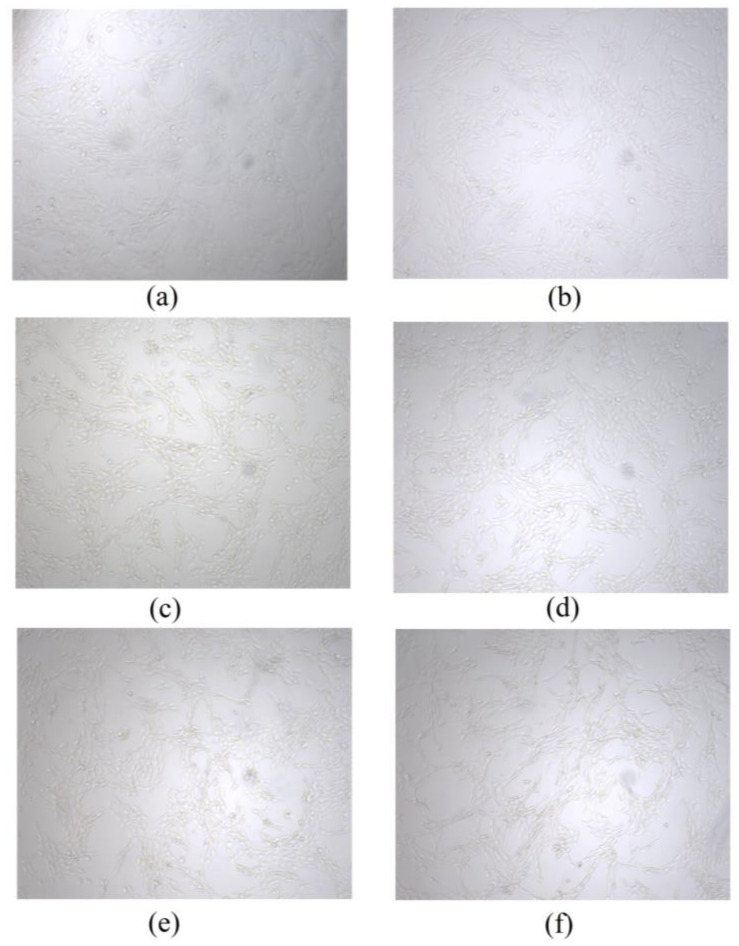
MC3T3-E1 cells morphology after adding different concentrations of BBC for cultivation. (**a**) 0 μg/mL; (**b**) 6.25 μg/mL; (**c**) 12.5 μg/mL; (**d**) 25 μg/mL; (**e**) 50 μg/mL; (**f**) 100 μg/mL.

The morphology of MC3T3-E1 cells is shown in Figure 14. Compared with the control group, no significant difference was observed in the morphology of MC3T3-E1 cells in different BBC concentration groups, and the cell morphology was uniformly spindle-shaped. All of the groups promoted the normal growth of cells. As the BBC concentration increased, the cell density of the experimental group was significantly greater than the control group. The cell density was the highest when the BBC concentration was 25 μg/mL. This result is consistent with the results of the aforementioned cell relative proliferation rate, indicating that BBC at the concentration of 25 μg/mL was most suitable for cell growth and safe as a biofunctional material.

## 4. Conclusions

BBC was successfully extracted from the swim bladders of black carp, achieving a high yield of 78.83 ± 0.13% (raw tissue dry mass), which was higher than that of bighead carp swim bladder collagen (59.00 ± 8.10%), grass carp swim bladder collagen (39.20%), and Gulf corvina swim bladder collagen. SDS–PAGE and UV spectroscopy confirmed that BBC is a type I collagen with a [α1(I)2α2(I)] structure. FTIR and XRD revealed that BBC retained a complete triple-helix structure. Secondary structure analysis conducted using infrared spectroscopy revealed that BBC comprised α-helix (38.65%), β-sheet (47.62%), β-turn (13.73%), and random coil (0%). Amino acid composition analysis revealed that the proline hydroxylation rate of BBC was 39.57%. The Td of BBC was 30.85 °C, which was significantly higher than that of deep-sea redfish skin collagen (16.1 °C), walleye pollock skin collagen (24.6 °C), and even exceeded human placental collagen (28.5 °C). The Tm value was higher than that of duck bone collagen (92.48 °C), duck skin collagen (86.22 °C), and duck tendon collagen (88.46 °C). Dynamic rheological results showed that within the frequency range of 0.01–10 Hz, the BBC solution exhibited elastic behaviour at a concentration of 5–20 mg/mL. The attainment of elastic properties of the material at low concentrations endowed BBC with distinct advantages in controlling food processing costs. At low temperature and low frequency, the solution exhibited a gel state dominated by elasticity, which met the requirement for system stability in the production of yogurt and semi-solid jams. Furthermore, under high-temperature and low-shear-rate conditions, the BBC solution retained its elastic characteristics. When BBC was applied in the processing of meat products such as sausages, the elastic characteristics enabled the maintenance of an elastic texture while reducing moisture loss during the cooking process. Steady-state tests confirmed that BBC exhibited shear-thinning characteristics, making it suitable as a raw material for cosmetics, such as eye creams and face creams. Functional properties analysis revealed that the EAI and ESI of BBC were greater than those of terrestrial collagen, including rabbit skin collagen, rabbit meat collagen, and rabbit ear collagen. The results of particle size distribution and CI collectively indicated that BBC exhibited the optimal emulsifying activity and emulsifying stability under acidic conditions, and the emulsions remained stable after 7 days of storage. The FC value of BBC was higher than defatted cashew protein, casein, and HBC 19 rice bran protein concentrate. The FS of BBC was higher than Basmati 370 rice bran protein concentrates, HBC 19 rice bran protein concentrate, and Basmati 386 rice bran protein concentrate. The WAC value of BBC significantly surpassed that of porcine skin collagen, casein, and cashew protein isolate. The OAC value of BBC surpassed that of chicken foot collagen, soy protein isolate, and cashew nut protein isolate. BBC exhibited excellent functional properties, while preventing potential safety risks associated with terrestrial animal proteins, thus providing a new pathway for the safe replacement of protein raw materials. Notably, BBC was non-toxic to MC3T3-E1 cells and promoted cell proliferation, enhancing its application potential in the biofunctional materials field. In conclusion, BBC exhibited significant advantages in extraction yield, thermal stability, rheological properties, functional properties, and biological properties, with promising industrial application prospects. Additionally, preparing high-value-added collagen using black carp swim bladder by-products as raw material enables the resource utilisation of waste, which is in line with sustainable resource utilisation and circular economy development. In future studies, the cross-linking process should be optimised to enhance thermal stability and mechanical strength; the improvement effects of enzymatic hydrolysis or chemical modification on functional properties should be evaluated; commercialised and large-scale extraction processes should be explored; and the practical performance of BBC in food, cosmetics, or biofunctional materials should be investigated.

## Figures and Tables

**Table 1:** The amino acid composition of BBC (results presented as residues per 1000 residues).

Amino Acid	BBC	Amino Acid	BBC
Ser	39	Leu	28
Gly	312	Phe	17
His	8	Hyp	55
Thr	31	Me	9
Glu	78	Val	30
Asp	58	Tyr	7
Ala	121	IIe	15
Arg	61	Hyl	6
Pro	84	Try	0
Cys	1	Imino acids *	139
Lys	41	Total	1000

* Imino acids refers to the sum of Pro and Hyp.

**Table 2:** Ostwald–de Waele model rheological parameters of BBC solutions at various concentrations.

Concentration (mg/mL)	*K*	*n*	*R* ^2^
5	1.512 ± 0.366	0.352 ± 0.013	0.994
10	13.707 ± 0.065	0.288 ± 0.003	0.999
15	40.342 ± 0.239	0.205 ± 0.003	0.999
20	76.822 ± 1.647	0.263 ± 0.012	0.997

**Table 3:** Ostwald–de Waele model rheological parameters of BBC solutions under various temperatures.

Temperature (°C)	*K*	*n*	*R* ^2^
10	17.331 ± 0.243	0.275 ± 0.008	0.999
20	14.068 ± 0.057	0.274 ± 0.002	0.999
30.85	1.841 ± 0.020	0.293 ± 0.006	0.997
40	0.088 ± 0.002	0.492 ± 0.015	0.986

## Data Availability

The original contributions presented in the study are included in the article. Further inquiries can be directed to the corresponding authors.
